# Identifying culturally acceptable cognitive tests for use in remote northern Australia

**DOI:** 10.1186/s40359-019-0335-7

**Published:** 2019-09-12

**Authors:** Deborah Rock, Ian R. Price

**Affiliations:** University of New England, Armidale, NSW 2351 Australia

**Keywords:** Aboriginal cognitive testing, Cultural acceptability, Remote Australia

## Abstract

**Background:**

A lack of culturally appropriate tests hampers accurate assessment of cognition in remote Australian Aboriginal communities. In Arnhem Land, this study employed a community consultation process to evaluate commonly used Western tests of executive function, memory, attention, and visuospatial function.

**Methods:**

An initial consultation process and a follow-up pilot study resulted in the rejection of some common tests, the development of new tests, and culturally adapted versions of others. In the subsequent 30-person main trial, adult Aboriginal volunteers were examined on nine tests, plus the Kimberly Indigenous Cognitive Assessment screen, and a brief literacy test.

**Results:**

Executive function, memory, and attention tests were found to group separately after an exploratory principal components analysis. Correlations between new tests and similar Kimberly screen items were not significant, but ceiling effects may be relevant. Six of 13 test scores were found to correlate with the literacy measure.

**Conclusions:**

A selection of cognitive tests were identified that Aboriginal people found culturally acceptable and engaging. In particular, Self-Ordered Pointing, Trail-Making, a verbal-switching task, and a new test “Which car?” show promise for further development. This work may contribute to the need for culturally appropriate cognitive testing in Aboriginal communities.

**Electronic supplementary material:**

The online version of this article (10.1186/s40359-019-0335-7) contains supplementary material, which is available to authorized users.

## Background

Accurate assessment of cognitive impairment is essential for ensuring people get the support they need. Effective assessment should integrate information from a range of sources including informant-reports and contextual information [1]. This is especially the case when working with Aboriginal populations [2, 3]. In such populations, cognitive tests do not provide sufficient information alone, but do provide meaningful information that supports assessment [4]. In remote parts of Northern Australia, Aboriginal people may be disproportionally exposed to factors impacting brain function, such as traumatic brain injuries, substance abuse, and gestational complications [4]. Compounding these challenges is the difficulty of diagnosing impairment in these populations due to a lack of suitable tools [5]; a situation requiring attention.

Although standard cognitive processes are similar for all humans, social and environmental differences influence the way cognitive processes are engaged, resulting in different patterns of abilities across cultures [6, 7]. Various studies have highlighted such differences for Aboriginal people. For example, compared to non-Aboriginal Australians, remote Aboriginal people differently approach spatial relationships [8]; and may rely more on visual skills when approaching numerical or memory tasks [9–12]. Identifying impairment requires understanding which skills are needed for normal function in a specific cultural context [4].

Few cognitive tests have been developed for assessing remote Aboriginal clients, consequently, clinicians in the Northern Territory (NT) use various Western tests, such as Wechsler scales, copying tasks, or progressive matrices [4]. Western tests typically assess abstract thinking, general knowledge, literacy, and numeracy - content taught in Western education systems. However, in many remote communities, there is inconsistent uptake of Western education, and literacy levels are lower than elsewhere in Australia [13, 14]. Western tests poorly reflect true abilities when used in low literacy, traditional populations [15, 16]. Scores on many types of tests are influenced by education and literacy; for example, working-memory [15, 16], and visual processing [17]. Even for supposedly culture-free tests, like the non-verbal Queensland Test, the scores are strongly linked to the level of Western education [2, 11]. The use of inappropriate tests can lead to misdiagnosis with serious consequences such as stigma or missed opportunities for support [3]. The Kimberly Indigenous Cognitive Assessment (KICA; [18]) was developed for remote Aboriginal populations, but only tests functions that deteriorate in dementia, such as verbal fluency, orientation, and recall. Although widely used across remote communities, it is only validated for use with older patients [4].

Feedback from NT clinicians [3], highlighted a lack of tools for testing younger adults, and a need to assess a broader range of abilities such as executive functions (EF), visuospatial functions, attention, abstract reasoning, decision making, and memory. These functions are typically impacted by alcoholism [19, 20] and volatile substance abuse [21, 22], both areas of concern in the NT [13, 23, 24].

According to previous research [3, 25] remote Aboriginal clients engage better with tests that can be intuitively understood, and which involve familiar stimuli. Tests in a game format also work well, especially if responses can be performed rather than verbalised. Tests requiring literacy, numeracy, timed tests, or overly long tests, are less appropriate. They recommend allowing for demonstrations, practice trials, and prompts if needed. Finally, if tests are portable, clients can be tested in familiar environments, making the experience less stressful, and the results more reflective of true abilities.

The current research took place in Maningrida in Arnhem Land. This remote community is home to around 2300 people from over nine language-clans [26]. The aim was to identify existing tests that might be culturally acceptable for all clans, and with community input, develop preliminary versions of new or adapted tests.

The project focused on tests of attention, visuospatial function, memory, and two aspects of EF: problem solving and flexibility. The research was conducted in three stages. Initially, consultations with senior community members was undertaken to identify culturally suitable tests. Community consultations were used to develop the KICA [3, 18]. Tests retained from this stage were trialled and refined in the second pilot testing stage. In the third stage, the approved tests were administered along with the KICA-Screen [27] and a brief English literacy test.

To summarise, the overall aim of the research was to find a selection of established cognitive tests that were culturally acceptable to Aboriginal people in northern Australia and with which they would willingly engage. Our analytic approach included examining the factor structure of the scores on the final set of tests to understand the nature of the cognitive constructs being assessed. We also examined the extent to which the scores from each test correlated with scores from the KICA that related to the same function. Finally, the extent to which the scores on the final set of tests correlated with an English literacy measure was examined.

## Methods

### Stage 1: consultations

#### Participants

Thirty working people from the community were invited to join consultation groups. Eleven women and five men participated, with one to four attending each meeting. Participants were Aboriginal teachers, health-workers and well-being workers, two were non-Aboriginal mental-health workers. All Aboriginal participants were multilingual, but principally identified with one language. Five people spoke Burarra, three Ndjebbena, two Kunwinjku, and one each spoke Nakara, Djinang, Mawng, and Luritja. All spoke reasonable English.

#### Procedures

The project was approved by the University of New England Human Research Ethics Committee, references HE16–278 and HE17–141. Data were collected in 2017.

Following the methodology described by [18], existing widely used cognitive tests were examined by the investigators for possible inclusion. Tests needed to be easy to use, adaptable, and low cost. These tests were then discussed with participants to determine which tests had potential for further development.

Consent was obtained from all participants before commencing. The consultation meetings were conducted in English. To protect anonymity, voices were not recorded. Suggestions were noted without names. Meetings lasted between 30 and 120 min. Some participants volunteered to attend multiple meetings. No one was paid, but refreshments were available.

Cognitive impairment was explained, then Western tests were presented one by one, with solutions demonstrated. Participants were invited to discuss whether each test would be understood by their clan. Any recommended modifications were noted, and adjusted versions were discussed with later groups. Participants were warned some tests were difficult. They were not asked to try them, but could if they asked to. The groups also considered which aspects of cultural knowledge could be tested to assess cognition. Promising suggestions were shared in subsequent meetings, to check if the knowledge was common to all clans.

#### Equipment

The following types of tests were discussed: Block Design; progressive matrices; Self-Ordered Pointing (SOP); Sorting Test; Tower of London; Design Fluency; Knox Cube (KCT); Trail-Making Test (TMT); a card-matching memory test (CMT); Simple-Figure Copy; Hooper Visual Organisation (HVO); Overlapping Figures; an Abstraction Test; a visual-attention test (VAT); Stick-Design Test (SDT); and two hand movement tests, Luria’s Three-Step, and Go/No-Go. The KICA-Screen was also discussed.

## Results

Participants rejected the progressive matrices, Tower of London, and Block Design. These were considered confusing and too reflective of non-Indigenous ways of thinking. Participants who tried them found them difficult, if not impossible to solve, even with detailed instructions. Another two tests were discarded because of difficulties with completion. The Abstraction Test required looking at two items and identifying the category they both belonged to, for example that bananas and oranges are fruit. More culturally familiar categories were tried, but some people kept identifying differences rather than categories. Design Fluency was approved by participants, but those who tried it were mostly unsuccessful. The decision to omit these two tests was based on a small number of observations.

The consultation groups approved several tests for the pilot stage after content modifications. Animals and supermarket items replaced abstract images in SOP. Stones and seashells replaced cards in the Sorting Test. For TMT, numbers and letters were replaced with lines and depictions of hand signals representing numbers. In Simple-Figure Copy, a line drawing of a turtle replaced the cube. For the visual organisation test, images of local animals replaced less familiar images. In the Overlapping Figures test, most objects were identifiable, but the watch and lamp were replaced by a fish and a chair. For VAT, the numbers were replaced by images of familiar animals and objects. Practice rounds were incorporated into several tests.

Several tests were approved by participants without changes: KCT, CMT, SDT, Luria’s Three-Step and Go/No-Go, although with a stipulation to perform the hand movement tests on a table, not the body. The KICA-Screen items were also all approved.

Four new tests were developed based on shared traditional knowledge: a verbal-switching task using sea and land sourced foods; a test to match animals and hunting implements; a test of knowledge of skin-group moieties; and a flexibility and problem-solving task called “Which Car” based on kinship avoidance. In total, 16 tests were approved for pilot testing.

### Stage 2: pilot testing

#### Method

##### Participants

To attract participants, the researcher distributed project information outside the local supermarket, and at a community drop-in centre. Sixteen women and six men volunteered for the pilot. Consultation participants were excluded, as was anyone known to have a cognitive or mental health problem. None of the participants spoke English as their first language, but all spoke some English. Eleven people spoke first-language Burarra, three Ndjebbana, three Kunwinjku, and one each spoke Nakara, Gurr-goni, Djinang, Kriol, and Mawng. Ages ranged from 21 to 49 years (*M* = 33.14, *SD* = 8.17).

##### Equipment

Stimulus materials were developed for each test and are described in the Results section. A draft version of the Main Trial score sheet was used.

##### Procedure

Testing took place in private. The researcher and the participant sat at a table across from each other. Consent was obtained before starting.

To be less onerous, the items were split into two groups of eight, A and B, each taking about 30 min. Most participants did one set, but two participants requested to do both. Sets A and B were alternated. Names were not recorded on scoresheets. Participants were invited to give feedback throughout, which the researcher noted by hand. Participants were not paid, but refreshments were available.

The pilot was used to fine tune tests, and to determine which would be used in the main trial. Judgements were based on participant feedback, and observations of which tests were performed appropriately.

#### Results

The following tests were retained for the main trial.

##### Hunting tools

**Fig. 1:**
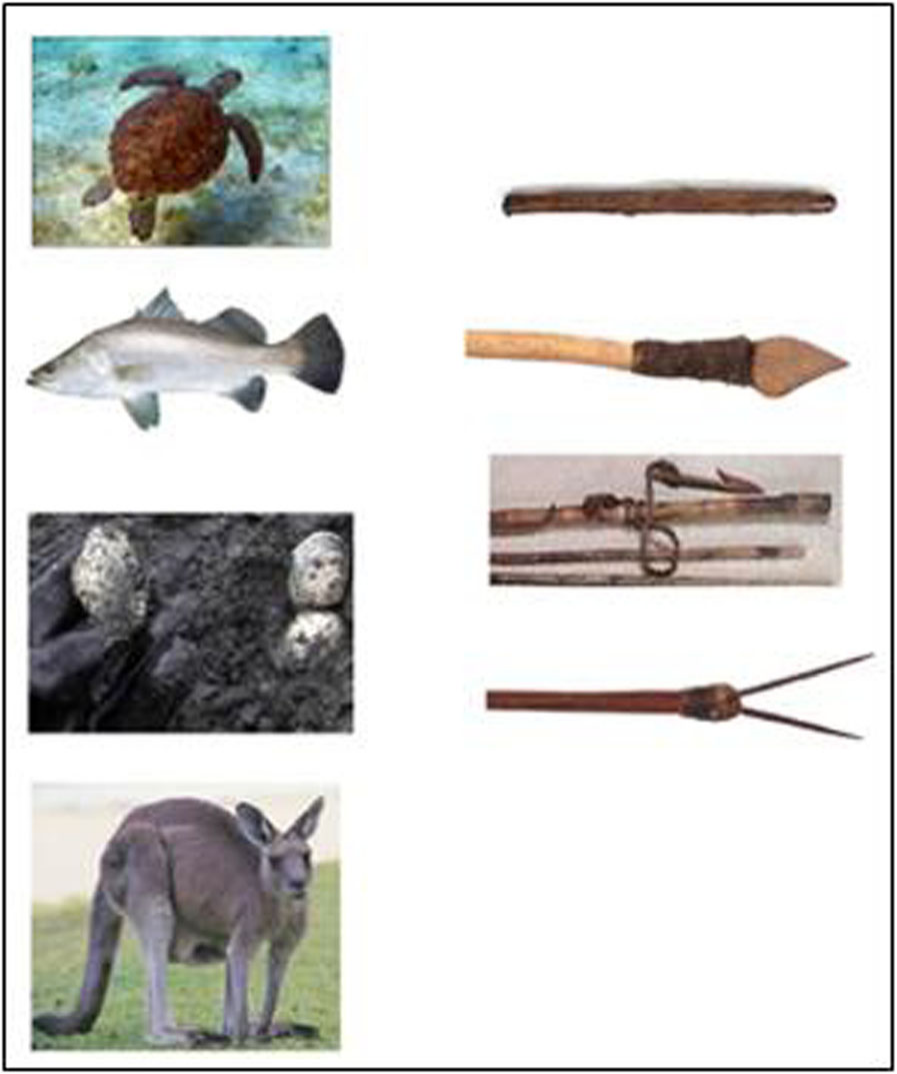
Hunting Tools test

On an A4 page are images of four frequently hunted foods and four hunting tools, see Fig. 1. The sheet is placed in front of the participant and the task is to match animals to the correct hunting tool, by pointing. Participants appeared to enjoy showing off their hunting knowledge and readily engaged with this test. Almost everyone answered correctly, although some participants appeared to reflect before answering. One person struggled on this task; testing was terminated with them as a precaution, and it was later discovered they had an intellectual impairment. This suggested possible value as a screen for the main trial.

##### Self-ordered pointing

**Fig. 2:**
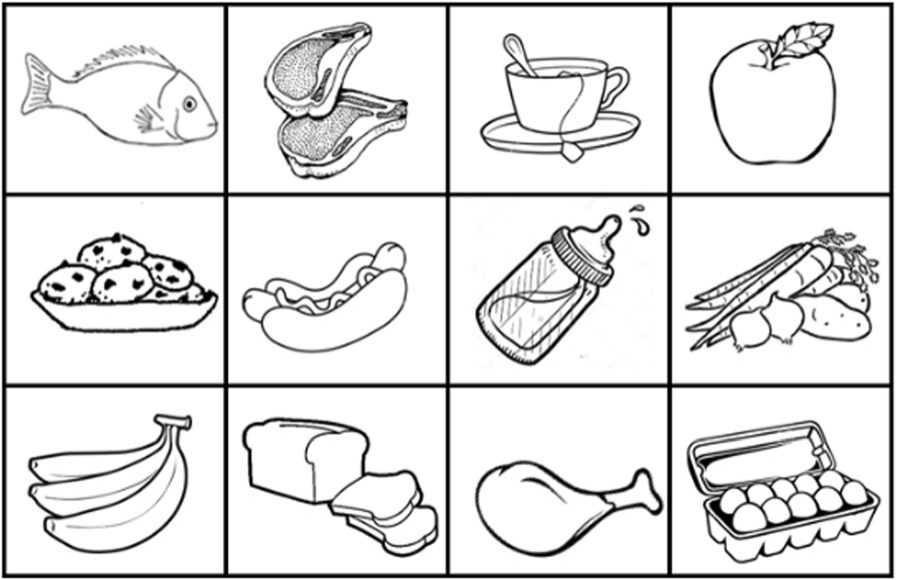
Self-Ordered Pointing, test array

On separate A5 pages are printed six arrays of six animals commonly found in the NT arranged in different orders (training phase) and twelve arrays of twelve common supermarket items arranged in different orders (test phase), see Fig. 2. Participants cross out a different object from each array, having to remember which object was crossed out on previous pages. Participants quickly understood what was required and engaged with the task.

##### Trail-making

**Fig. 3:**
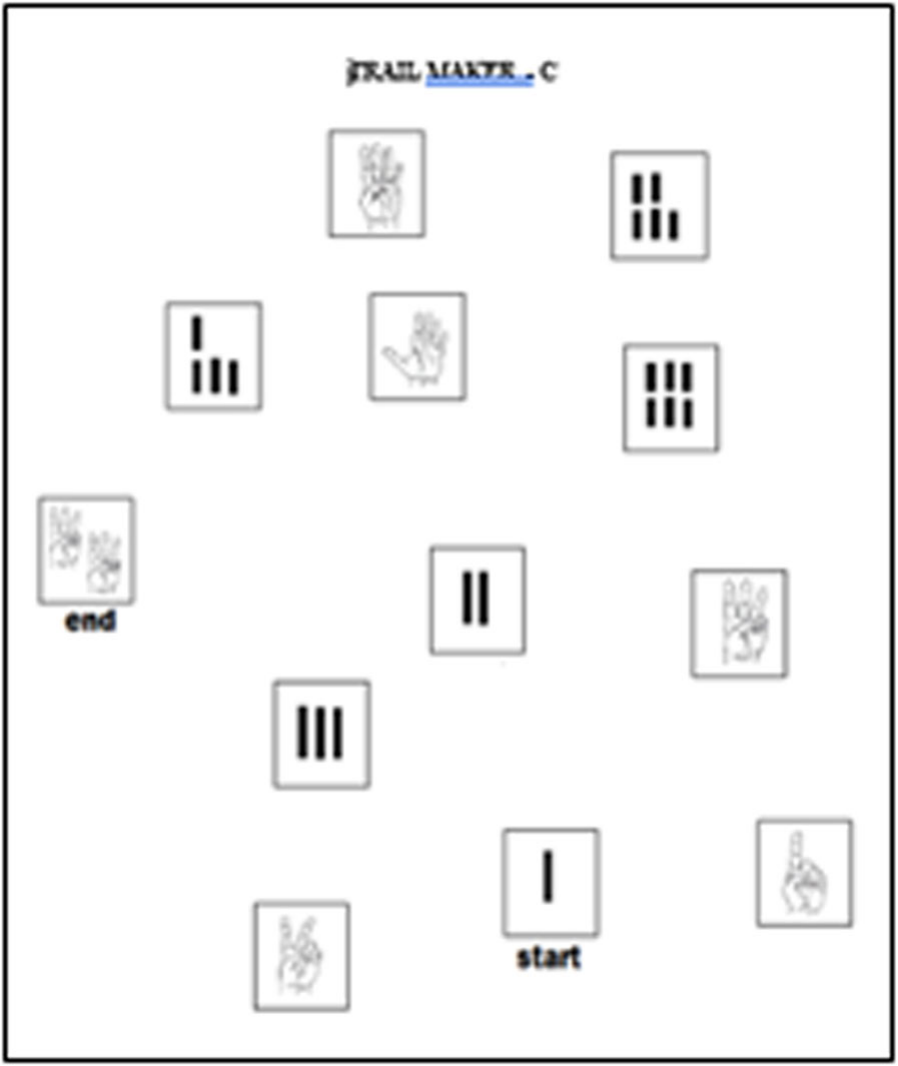
Trail Making test

A short, adapted version of Trail-Making which replaces numbers with images of hands and replaces letters with lines. There are 6 images to sequence in parts A and B, and 12 images to sequence in part C, which alternates hands and lines, see Fig. 3. Everyone identified the correct numerical sequences using the adapted format. About half made some errors during the switching, but appeared to understand the task. One participant found the switching “a bit confusing” but another said: “it’s alright, it wakes my mind up”.

##### Card-matching

**Fig. 4:**
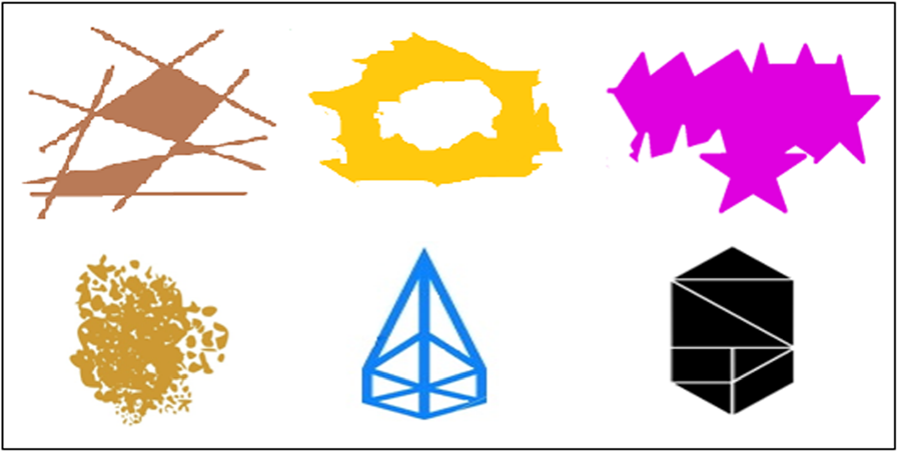
Some of the abstract images used in the Card Matching test

Laminated cards 10 cm × 7 cm were printed with one abstract image each (Fig. 4). There are two copies of each image. Five or eight cards face down on the table are turned over and back one at a time; identical cards are used to test memory of locations. Everyone quickly understood the task and engaged readily, often smiling while attempting it. Many participants did poorly with eight cards, suggesting that number is excessive.

##### Visual-attention

**Fig. 5:**
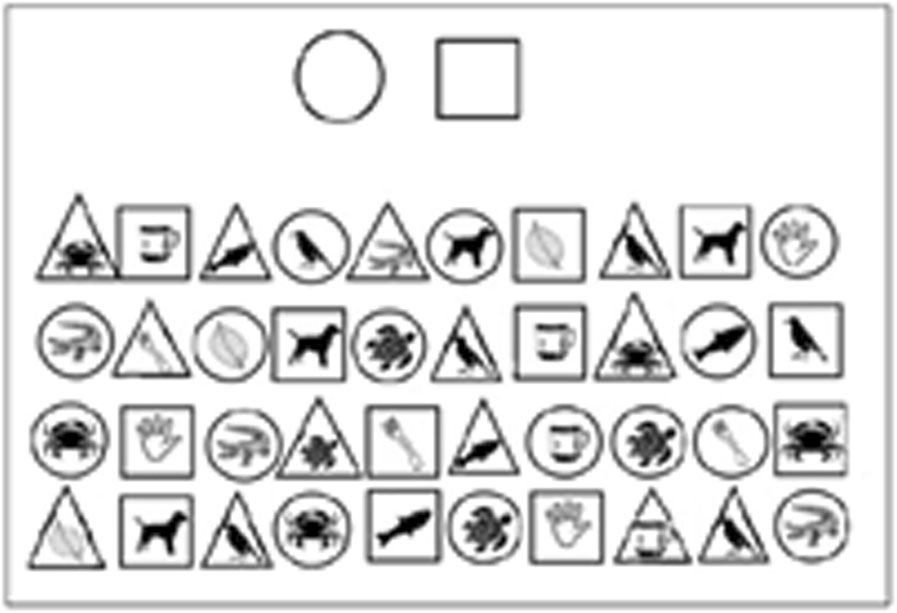
Visual Attention Test

Participants are presented with a printed page containing rows of squares, triangles, and circles. Each shape has a common object depicted inside it. Participants “read” along the line, only naming objects inside the shape or shapes specified at the top of the page, see Fig. 5.

Everyone recognised the objects and performed the task in the correct manner. There were errors, but they seemed to reflect attention lapses rather than task comprehension issues.

##### Stick-design

**Fig. 6:**
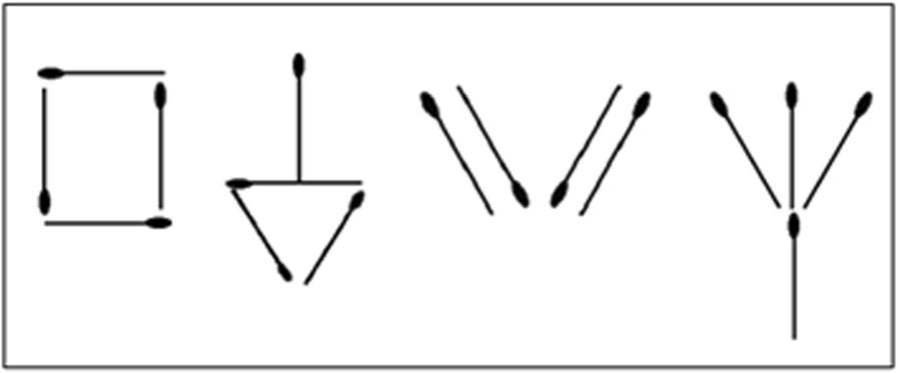
Stick Design shapes

Use matchsticks to copy shapes, see Fig. 6. The task was quickly understood and appeared enjoyable.

##### Which Car

**Fig. 7:**
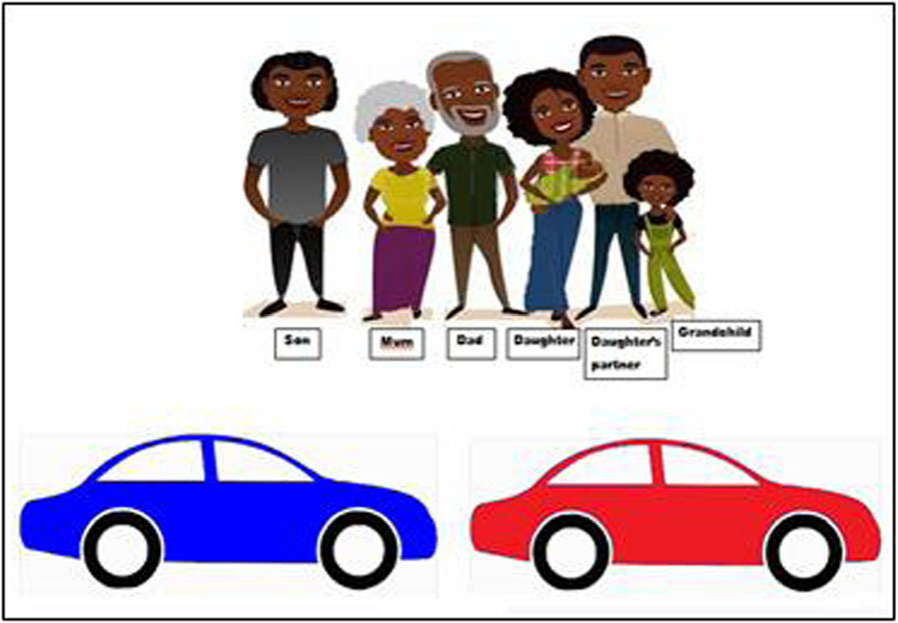
Which Car Test

Find multiple ways to allocate an extended family to two cars, while respecting traditional avoidance relationships. For example, a mother-in-law and son-in-law are considered “*poison cousins*” and must be kept apart, likewise brothers and sisters. When deciding which family members to put into which car, these avoidance relationships must be respected. A printed page is provided representing the family and the two cars. A marker pen is used to draw lines between each person and the appropriate car, see Fig. 7. The requirements took longer to explain than other tests, but performances indicated everyone understood. Comments were overwhelmingly positive, including: “this game is good, this is our culture”, and “this is really what we do”. The pilot sought four distinct arrangements, but that seemed too challenging, three may be enough.

##### Sea-land verbal-switching

**Fig. 8:**
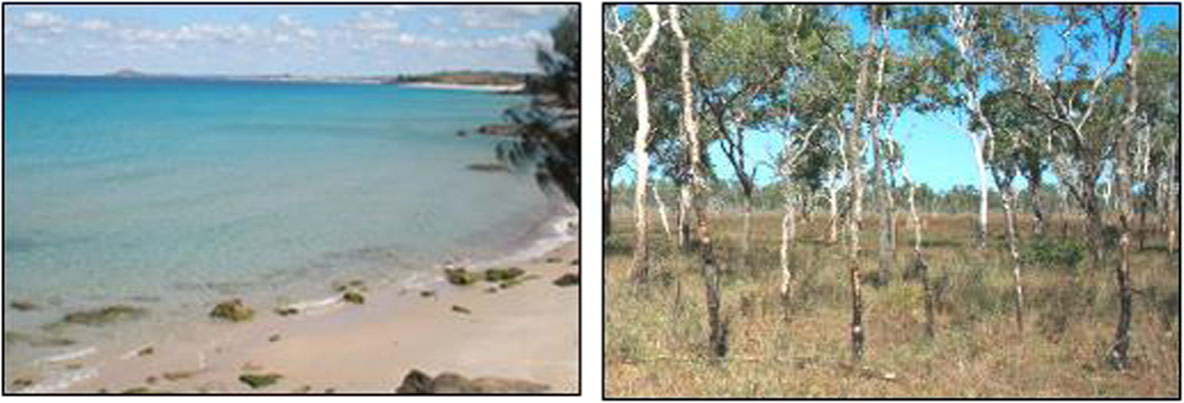
The sea (left) and land (right) images used to facilitate the verbal switching task

Name a food from the sea, then a food from the land; repeat five more times. This is like D-KEFS Verbal-Fluency Category Switching [28], but with more familiar categories, with images to guide the task, and recording the time based on a fixed number of repetitions. Participants switched categories better when they had the images as prompts, see Fig. 8. Comments included: “*it makes my brain work”*.

##### Knox cube

The tester touches a set of cubes in a specific order, the participant must repeat the sequence. The test’s simple instructions were easily understood. Those who had trouble with this test also had difficulties with CMT.

##### Discarded tests

The following tests were not used in the main trial. Some because they were unsuitable, others to limit the size of the trial.

##### Moieties test

Skin-names belong to one of two moiety groups. Identify the correct moiety for eight different skin-names. Some participants found this easy, others needed time to reflect, but most answered correctly for all eight. However, difficult pronunciations made test delivery challenging.

##### Sorting test

Sort a group of shells and stones into two groups in different ways. Only one person identified all the possibilities. Notably, 75% incorrectly proposed equivalent instead of distinct groups, for example two large and two small objects in each group. This raises questions about the test’s suitability for this population.

##### Simple-figure copy

Copy a turtle line drawing. The turtle idea was approved, but 80% were unsuccessful copying the hexagon pattern in the shell. One said the hexagon was culturally unfamiliar. The design needs further consultation.

##### Hooper visual organisation

Identify animals from rearranged pieces. The task was completed quickly suggesting this set of images was too easy.

##### Overlapping figures

Identify eight superimposed line drawings. This was popular, but several people mistook the chair for a table so the design needs adjustments.

##### Go/no-go

Tap on the table or refrain, in response to the tester tapping. After time to practise, everyone mastered this task, although one said: “I had to concentrate”. This was only discontinued to reduce the high number of tests in the main trial.

##### Luria’s three step

Copy a sequence of three hand movements. After a practise round, all participants correctly managed the task. Participants found it acceptable but boring.

### Stage 3: Main trial

#### Method

##### Participants

To recruit participants, the researcher distributed project information at the supermarket and the community centre, as per the pilot. In addition, the Community Development Program let the researcher give information to their workers. Twenty-three women and seven men volunteered. Anticipating a medium effect size, this was less than the minimum number required for adequate power. A G*Power analysis [29] for a one-tailed correlation, with α = .05, and power of .8, indicated 67 participants were needed. Participants from Stages 1 and 2 were excluded, as was anyone known to have a cognitive or mental health problem. One potential participant was excluded because they could not do the Hunting Tools test. Anyone who spoke English as a first language was also excluded, nevertheless, all participants spoke some English. Fourteen spoke Burarra as their first language, five Ndjebbana, three Gunartpa, three Nakara, two Kriol, and one each spoke Gurr-goni, Rembarrnga, and Tiwi. Ages ranged from 21 to 50 years (*M* = 34.1, *SD* = 9.60).

##### Equipment

The KICA-Screen was used [27]. This test has 10 items, most are answered verbally, but one, a test of Executive Function, involves copying a pattern of alternating circles and crosses. The screen has been validated for assessing cognitive difficulties in older Aboriginal people. It has high inter-rater reliability, and over 95% sensitivity for detecting dementia. It is widely used to assess cognition across many ages [4] but is not specifically validated for that purpose. No other validated test was available. To simplify processing for this study, the KICA-Screen was divided into subgroups. KICA-Memory included items 7 and 8 covering free and cued-recall. KICA-Verbal was item 5 assessing verbal fluency. KICA-EF refers to item 7, the EF test. KICA-General included the easiest items: 1, 2, 3, 4, 6, and 10 which cover orientation, verbal comprehension, naming objects, and praxis.

Nine tests from the pilot study were included in this trial. An ad-hoc literacy test was added to these. It involved reading eight common words drawn from 2nd to 5th grade word lists: hat, play family, friend, tomorrow, question, language, and banana. Years of education, a common alternative, was considered unreliable due to high rates of school non-attendance [30].

For timed tests, a stopwatch application on a Samsung Galaxy mobile phone was used. IBM SPSS Statistics 25.0 was used for the statistical analyses.

##### Procedure

Testing took place in private spaces, with the researcher and the participant sitting at a table across from each other. Before beginning, consent was obtained, with an interpreter’s assistance if required. The interpreter did not participate in testing. One researcher (DR) conducted all testing. DR had lived in the community for 3 years and had worked as a research assistant on qualitative and quantitative projects during this time.

The presentation order was varied to avoid order effects. The KICA-Screen was presented either first or last, and this was alternated. Other tests were divided into groups A, B, and C, with the presentation order rotated: ABC, BCA, CAB. Delivery took 45–60 min. Iced water was available throughout. Short breaks were permitted.

For the KICA-Screen, the test’s standard script was used. For other tests, scripts trialled in the pilot were used. Standard scoring systems were used where available, otherwise scoring systems developed and refined during the pilot were applied. Responses were recorded directly onto scoresheets (see Additional file 1). These were held by the researcher and not visible to participants. No identifying information was noted on the scoresheets. Participants were welcome to give feedback throughout, which the researcher noted by hand. Participants were not paid.

#### Results

KICA-General scores were excluded from all analyses as only one person made one error, a considerable ceiling effect. For Trail-Making, two scores were used: B-A, which is the difference in speed between the alternating (B) and non-alternating (A) tasks, considered a measure of switching ability [31]; and accuracy on the alternating task, derived from the error rate, this assesses EF and working memory [32].

As the assumptions of normality and linearity were not met for some items, Spearman’s rho (*r*_*s*_) was used for all correlations. Missing scores were judged to be random. Missing data came from two participants who did not finish testing, and some randomly missed speed scores. These were all excluded pairwise from correlations. There were two univariate outliers on two tests. The mean and *SD* were recalculated without them, and the outliers were replaced by the equivalent of *z* = 3.29. All speed scores were standardised, then reversed by subtracting from four. This does not change the strength of correlations [33], but meant that scores representing higher ability, such as the number of correct answers and faster task completion, were expected to correlate positively.

##### Principal components analysis

A principal components analysis involving all 16 items was judged unviable due to the size of the group and the range of cognitive functions drawn on. Therefore, an analysis was performed on only the 11 items pertaining to memory, attention and EF, as listed in Table 1. Due to low intercorrelations with all other items, the memory items from the KICA were excluded from this analysis.

**Table 1:** Factor Analysis of Memory, Attention, and Executive Function Items

Tests	Factor 1	Factor 2	Factor 3	Communalities
Card-Matching	.78			.67
Self-Ordered Pointing	.73	.42		.72
Knox Cube	.72		.32	.63
Visual-Attention, accuracy			.88	.86
Visual-Attention, speed	.75		−.42	.79
Which Car	.70			.58
Trail-Making, accuracy	.71		.33	.62
Trail-Making, B-A	.31	.56		.42
Sea-Land Verbal-Switch, accuracy		.82		.73
Sea-Land Verbal-Switch, speed		.79		.64
KICA-EF		.67		.52
Variance explained after rotation	31.71%	21.99%	11.60%	

*Note.* Factor loadings < .3 are suppressed

For the analysis, eight missing SLVS-speed scores were replaced with standardised SLVS-accuracy scores as they correlated at *r*_*s*_ = .55. This did not greatly change the loadings. Missing data from partially completed tests were excluded listwise. Mahalanobis Distance scores confirmed that no multivariate outliers remained. A Shapiro-Wilks test indicated that some of the items were not normally distributed, however visual examination of the Normal Q-Q plots showed this was only minor. The analysis went ahead as exploratory factor analyses can tolerate violations of normality [34].

The reduced set of 11 items was deemed suitable for principal components analysis, KMO = .79. Eigenvalues supported the extraction of three factors, which explained a total variance of 65.29%. Orthogonal rotation provided the most interpretable outcome. The results are in Table 1.

A tentative interpretation is that Factor 1 relates to working-memory as all of the memory items loaded heavily on this factor as did items requiring information to be held and manipulated. Factor 2 is interpreted as executive control as items related to organising information and cognitive switching loaded on this factor. Factor 3 is interpreted as relating to attention.

##### Correlations with KICA and literacy

There were no significant correlations between KICA-Memory and any memory items; the largest association was a weak correlation with CMT, *r*_*s*_ = .28 (ns). Correlations between KICA-EF and EF items are in Table 2. Seven tests significantly correlated with the literacy test, these are all noted in Table 3.

**Table 2:** Spearman Correlations Between KICA-EF and Executive Function Test Scores

Executive function tests	KICA-EF
Sea-Land Verbal-Switching, accuracy	.46** (29)
Sea-Land Verbal-Switching, speed	.43* (21)
Which Car	.32* (29)
Trail-Making, accuracy	.28 (28)
Trail-Making, B-A	.27 (28)

*Note.* The number of participants is indicated in parentheses

*p < .05. **p < .01. One tailed, positive correlations only

**Table 3:** Tests that Significantly Correlated with Literacy^b^

Tests	Literacy
KICA-EF	.61*** (29)
Stick-Design accuracy	.55*** (29)
Visual-Attention, speed	.46** (28)
Card-Matching	.41* (29)
Sea-Land Verbal-Switching, speed	.37* (22)
Self-Ordered Pointing	.36* (29)
Hunting Tools	.32* (30)

*Note*. The number of participants is indicated in parentheses

^*b*^The lowest correlation was with Which Car, *r*_*s*_ = .14

*p < .05. **p < .01. ***p < .001. One tailed, positive correlations only

## Discussion

The community feedback received during test development provided valuable insights into the types of tests remote Aboriginal people find acceptable. This information is important if neurocognitive tests are to be used with appropriate specificity and sensitivity in Aboriginal communities.

### Community feedback

The community rejected several tests, including the Tower of London, Block Design, progressive matrices, and the Sorting Test. The skills required to solve these tests align poorly with the knowledge and cognitive skills relevant for this cultural context. This outcome supports what other studies have found when working with traditional peoples. Indigenous South Americans found Block Design and Sorting Test virtually impossible [15], suggesting the tests lacked cultural relevance. Raven’s Matrices failed to correlate with tests of traditionally relevant knowledge in rural Africa [35]. These non-verbal tests may be acceptable for Aboriginal people with substantial Western education, but in this study, community members found them perplexing. This is concerning as they continue to be used, yet results are unlikely to reflect true ability. The preference for tangible over conceptually abstract tests [25] was seen in the approval of other tests, including Go/No-Go, CMT, SDT, and KCT.

Notably, the consultation process produced culturally acceptable ways to adapt two number based games for non-numerate clients. In TMT, culturally familiar hand signals and dashes replaced the numbers one to six. This was successful as pilot and main trial participants followed the sequences correctly. For VAT, numbers were not intrinsic to the task so were replaced by common objects which also produced the desired responses. These approaches to adapting number-based tests for remote Aboriginal populations may be useful for future test developers.

Overall, feedback to the final set of tests was positive. The use of familiar objects, practice sessions and shorter tasks worked well, supporting previous recommendations [3]. Comments included: “it’s alright, not too much balanda *(non-Indigenous)* way”; and “it helped me to focus and to bring back my memory”. Some participants noted that they felt happier afterwards, and several suggested using the tests for wellbeing.

### Results of analyses

The principal components analysis showed that the 11 test scores could be described by three factors that explained 65% of the overall variance. The factors were interpreted as working-memory, executive control, and attention. The memory items all loaded predominantly on the working-memory factor, with KCT loading on both memory and attention, consistent with earlier research [36]. The SLVS scores, KICA-EF scores, and the adapted TMT B-A scores loaded together on the executive control factor. TMT B-A is considered a relatively pure test of cognitive-switching [31]; SLVS was proposed as a verbal way to assess switching. The similar loadings between the two tests is encouraging, given they use different modalities to test the same function. Several tests loaded on two factors in line with earlier studies. The adapted SOP loaded on memory and executive control, agreeing with [37]. TMT-accuracy loaded on working-memory and attention, agreeing with [32]. The VAT-accuracy score loaded on the 3rd factor, attention, in line with expectation and suggesting it may have some potential for this population. However, the VAT-speed score loaded negatively on attention, and higher on working-memory. Earlier research showed that processing is directly linked to working-memory capacity [38] which may underlie this result. Overall, the factor analysis showed that there was not a clear simple structure. This probably reflects the fact that many tests are not pure tests of just one mental function, but rather draw on several functions. The loadings were consistent with those seen in earlier studies suggesting that the adapted tests may perform similarly to their original versions.

There was little relationship between the new items and similarly labelled KICA items. None of the memory items, CMT, SOP, or KCT, significantly correlated with KICA-Memory. KICA-Memory assesses delayed recall whilst the new items assess immediate recall; nevertheless, in healthy populations they should correlate [39]. KICA-EF only correlated significantly with some EF items. The results do not support the idea that the current test items assess the same cognitive ability as the corresponding KICA items. A likely reason for this is the ceiling effect seen on most items. The healthy non-clinical nature of this sample compared to the sample from which the KICA-Screen was developed meant few errors were made. More research is required into comparisons between the tests used in this study and the KICA.

There were mixed results on the final analysis regarding correlations with literacy. We used an ad-hoc literacy test and so results are not definitive, but give an indication of where literacy might be relevant. A highly significant correlation between literacy and SDT-accuracy supported the claim [40] that people with low literacy have problems managing visual information compared to more literate people. A strong correlation between literacy and VAT-speed was consistent with [41] who found that literate participants are faster on visual scanning tasks. The non-significant correlations with CMT, VAT-accuracy, both TMT scores, and SLVS-accuracy are encouraging, but are not conclusive as the study was underpowered, and many correlations were performed, increasing the risk of errors. Finally, the very low correlation with the kinship test “Which Car” suggests this problem-solving task may use culturally relevant skills that are unrelated to education. This is worth further exploration.

### Limitations

Several factors limit the conclusions that can be drawn from this study. As one researcher conducted all testing, expectation bias cannot be ruled out. Also, the KICA, which was included for comparison purposes, was insufficiently sensitive for this healthy sample. Consequently, the results should be used to give some indication of future potential of the final set of tests but much work is required to establish their validity. Twelve clans from Maningrida participated in this research suggesting reasonable participant diversity and hence generalisability to the wider Aboriginal community in Northern Australia. Nevertheless, Maningrida is a coastal community, and this is reflected in test content. Future research will need to consider each test’s acceptability elsewhere, and whether different versions are needed for different regions. Also regarding the sample, as participants self-selected for this study, the sample may be biased because the researcher was non-Indigenous. Not all Maningrida residents are relaxed with non-Indigenous people, consequently, those who volunteered may have had more Western contact and education than is typical for this region. A final issue was the limited time frame for conducting the research. Unpredictable events, such as deaths in community, greatly reduced testing opportunities and affected group sizes, especially for the pilot. The reduced opportunity to fine tune the tests ultimately affected which tests could be included in the main trial. A longer research period may have led to different conclusions.

## Conclusions

This study represented initial work on the suitability of various neurocognitive tests in a healthy sample of Aboriginal participants. Going forward, these new and adapted tests will need rigorous assessment of their reliability, particularly test-retest and inter-rater reliabilities, and their ability to distinguish healthy function from impairment. Some of these tests showed potential and hopefully will be taken down that path, but acceptability and engagement by Aboriginal people is a necessary first step before psychometric properties can be established. Factor loadings suggest that these versions of Trail-Making, Sea-Land Verbal-Switching and Self-Ordered Pointing may be worth considering for this population. The cognitive constructs being assessed by the kinship based test “Which Car” need more research, but may be worth pursuing as a culturally relevant way to assess problem solving skills. Furthermore, there were piloted tests that were not trialled, but which appeared to be acceptable. Amongst these was Go/No-Go which tests inhibition [42], and Overlapping Figures, a simple way to detect problems in visuoperception [43].

The different cognitive skills of remote Aboriginal people make cognitive testing in this population challenging. Nevertheless, this study reminds us that by working closely with communities, it is possible to identify culturally suitable tests, and to adapt tests to be suitable. Initial results suggest some adapted tests may tap into similar processes as their mainstream versions, which is promising. Maningrida community was supportive of this study and would like to see more culturally appropriate tests used for assessments. Establishing the reliability and validity of new or adapted tests can take years, but the need for accurate assessments is high. It is important to see this work continue.

## Additional file

Additional file 1: Scoresheet used in the Main Trial. (DOCX 48 kb)

## Data Availability

Data can be found at https://hdl.handle.net/1959.11/27366

## Abbreviations

CMT: Card-Matching Test
EF: Executive function
HVO: Hooper visual organisation
KCT: Knox cube test
KICA: Kimberley indigenous cognitive assessment
NT: Northern territory
SDT: Stick design test
SOP: Self-Ordered pointing
TMT: Trail-Making test
VAT: Visual attention test
