# Supplementary material for: Identifying culturally acceptable cognitive tests for use in remote northern Australia
Source: BMC Psychol. 2019 Sep 12;7:62. doi: 10.1186/s40359-019-0335-7 (PMC6740030; doi:10.1186/s40359-019-0335-7)
Supplement: Supplementary file 1 — Scoresheet used in the Main Trial. (DOCX 48 kb) [file 40359_2019_335_MOESM1_ESM.docx]

Additional file 1

Scoresheet used in the Main Trial.

The numbers refer to the tests described in the Results section of the pilot testing stage of the research.

**1 Hunting Tools**

Digging stick for clam Shovel spear for kangaroo
Harpoon for turtle Forked spear for fish

Scoring: 1 point for each correct (max 4) Total ______/4

**2 Self-Ordered Pointing (SOP)**

*An error is when the same item is crossed off more than once.*
6 items Trial 1 Number of errors: _____

Trial 2 Number of errors: _____ Score: _____

12 items Trial 1 Number of errors: _____

Trial 2 Number of errors: _____ Score: _____ Total ______/4

Scoring: 6 item array All correct in 1^st^ or both trial 2 points

All correct only in 2^nd^ trial 1 point

Errors in both trials 0 points

12 item array All correct in first trial 3 points
 All correct only in 2^nd^ trial 2 ½ points

Best trial 1 error 2 points
 Best trial 2 errors 1 point

Best trial 3+ errors 0 points

**3 Trail-Making (TMT)**

*Record errors and speed.*

**Timing**

Hands only Time: _________________(A)

Lines only Time: _________________(B) A + B: ____________ (C)

Alternating Time: _________________(D) D – C: _____________ (E) *note this*

*Only note errors on the alternating task. Ignore self-corrected errors.*

Errors on alternating task: _______________

Scoring No errors 2 points
 1 error 1 point
 2+ unrelated errors 0 points Total: ______/2

**4 Card-Matching Task (CMT)**

Five cards Trial 1 Number of errors: _____

 Trial 2 Number of errors: _____

*Optional 3^rd^ trial* Number of errors: _____
 *Offer a 3^rd^ trial if it appears they are performing below ability and the participant wishes to.*

Seven cards Trial 1 Number of errors: _____

 Trial 2 Number of errors: _____

Scoring five cards 2 trials no errors 3 points

1 trial no errors 2 points

3 correct/1 pair swapped in best trial 1 point Score for 5 cards: --------/3

Scoring seven cards No errors in either trial 4 points

Best trial 5 correct/1pair swapped 3 points
Best trial 4 correct/3 cards swapped 2 points

More than 4 errors 0 points Score for 7 cards ______/4

Total: _______/7

**5 Visual Attention Task (VAT)**

*An error is counted for: Missed object; wrong order; named a wrong object.*

**Circles only**

Bird __ dog __ hand __ crocodile __ leaf __ turtle __ fish __ crab __ crocodile __ cup __ turtle __ fork __ crab __ turtle __ crocodile __

Time taken to complete: _____________ Total errors: __________

Circles score No errors 2 points
1 error 1 point
2 + errors 0 point Circles only score: ____/2

**Circle & squares**

Hand__ dog__ leaf__ dog__ bird __cup __ bird__ fish__ cup__ turtle__ dog__ leaf__ crocodile__ crab__ fork__

turtle__ cup__ fork__ crocodile__ hand__ crab__ crocodile__ hand__ turtle__ fish__ crab__ dog__

Time taken to complete: _____________ Total errors: __________

Circles + squares score No errors 3 points

1 error 2 points
2 errors 1 point
3 + errors 0 points Circles + squares score: ____/3

Total speed for both tasks: _________________ Total score: _____/5

**6 Stick-Design Test (SDT)**


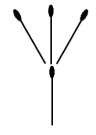

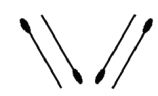

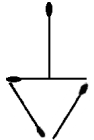

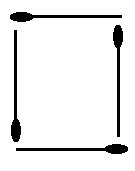


____ ____ ____ ____

Time taken to complete: ________________

Scoring: No errors 3 points per design (max 12)

Correct but upside down subtract 1 point per design

Any matches wrong way around subtract 1 point per design

Total score:_____/12

**7 Which Car**

Record: Blue car: B

Red car: R

|  | Round 1 | Round 2 | Round 3 | Round 4 |
| --- | --- | --- | --- | --- |
| Son (brother) |  |  |  |  |
| Mum |  |  |  |  |
| Dad |  |  |  |  |
| Daughter (sister) |  |  |  |  |
| Daughter’s partner (son- in-law) |  |  |  |  |
| Grandchild |  |  |  |  |

Rules: Cannot have mother & daughter’s partner together. Cannot have son & daughter together.

Scoring 3 correct & distinct arrangements in 3 trials 4 points
 3 correct & distinct arrangements in 4-5 trials 3 points
 2 correct & distinct arrangements 2 points
 1 correct arrangement 1 point

Total score ___/4

**8 Sea-Land Verbal-Switching (SLVS)**

*An error is counted for a food: repeated, named out of order, too similar (e.g. sea mud mussel and land mud mussel).*

Sea - Land ____ Sea - Land ____ Sea - Land ____

Sea - Land ____ Sea - Land ____ Sea - Land ____

_________________________________________________________________________________

_________________________________________________________________________________

Total number of errors: _________________ Time taken to complete 6 pairs: _______________

Scoring: All correct 3 points
 Errors Minus a ½ point for each error Total score _____/3

**9 Knox Cube Test (KCT)**

*Cross if unsuccessful, tick if successful.*

|  | 1st | 2nd | 3rd | 4th | 5th |
| --- | --- | --- | --- | --- | --- |
| A |  |  |  |  |  |
| B |  |  |  |  |  |
| C |  |  |  |  |  |
| D |  |  |  |  |  |
| E |  |  |  |  |  |

Total number of attempts: _________

Scoring Score seven if all five games are correctly copied first try.
Minus ½ a point for each additional attempt.
Minus an additional ½ point if they fail to copy a game correctly in the allotted number of attempts.

Total score: _______/7

**10 Literacy**

Hat Play

Family Friend

Tomorrow Question

Language Banana

Scoring: All correct: 4 points

6-7 words correct: 3 points

3-5 words correct: 2 points

1-2 correct: 1 point

Declined 0 points
